# Hepatitis E Virus Variant in Farmed Mink, Denmark

**DOI:** 10.3201/eid1912.130614

**Published:** 2013-12

**Authors:** Jesper S. Krog, Solvej Ø. Breum, Trine H. Jensen, Lars E. Larsen

**Affiliations:** Technical University of Denmark, Frederiksberg, Denmark (J.S. Krog, S.Ø. Breum, L.E. Larsen);; Aalborg University/Aalborg Zoo, Aalborg, Denmark (T.H. Jensen)

**Keywords:** zoonoses, viruses, Denmark, hepatitis E virus, HEV, hepatitis, real time RT-PCR, mink, neovison vison

## Abstract

Hepatitis E virus (HEV) is a zoonotic virus for which pigs are the primary animal reservoir. To investigate whether HEV occurs in mink in Denmark, we screened feces and tissues from domestic and wild mink. Our finding of a novel HEV variant supports previous findings of HEV variants in a variety of species.

Hepatitis E virus (HEV, family *Hepeviridae*) is a main cause of acute liver inflammation in humans. It is a nonenveloped RNA virus with a positive-sense genome of ≈7.2 kb. In 1997, HEV was discovered in pigs (*1*), and several studies have since shown that HEV is endemic in pigs and that pigs probably are a major animal reservoir. HEV traditionally had been divided into 4 primary genotypes (G1–G4). G1 and G2 have been found only in humans. G3 has been found globally in a wide range of mammals, including humans, pigs, deer, rabbits, and mongooses. G4, like G3, has an animal reservoir and has been found in humans, pigs, and wild boars (*2*).

Along with the human and porcine variants, avian HEV (aHEV) has been characterized. It is widespread globally and has been proposed to comprise 3 genotypes (*3*). Since 2010, several novel HEV variants have been described in red foxes, cutthroat trout, rats, bats, and ferrets (*4*–*8*). All new variants clearly differed from HEV G1–G4, aHEV, and each other. HEV is highly prevalent among pigs in Denmark; 92% of herds are seropositive, and ≈50% of investigated herds had pigs positive for HEV RNA (*9*). Because HEV is highly prevalent in swine in Denmark, animals fed offal from Danish slaughterhouses will be exposed to HEV. Production of mink fur is a major industry in Denmark, and mink are routinely fed a mixed diet, which often includes swine offal. Inappropriate heat-treated swine offal has previously been shown to be the source of swine-related influenza A virus infection in mink (*10*,*11*). Thus, we aimed to investigate whether mink in Denmark are infected with HEV G1–G4 or other HEV variants by screening fecal and tissue samples from domestic and wild mink.

## The Study

Initially, we screened 85 fecal samples collected during 2006 through mid-2012 from farmed mink by nested PCR; a broad panel of HEV variants was detected (*6*). One sample was positive, and subsequent sequencing and phylogenetic analysis showed that this virus represented a new HEV variant. To screen more samples for this new virus, we developed a specific real-time reverse transcription PCR (RT-PCR) (Technical Appendix). The initially tested 85 fecal samples and an additional 233 fecal samples from farmed mink, together with liver and fecal samples from 89 wild mink, were tested with this new and more sensitive assay. We identified 4 positive samples, all from farmed mink. In addition, screening with an HEV real-time RT-PCR (*9*) specific for G1–G4 found none positive. The HEV-infected mink were all submitted for diagnostic examination; all had histories of diarrhea in the herd. Three submissions were from herds having mink enteritis virus. Lipidosis, Aleutian mink disease virus, and catarrhal enteritis also were diagnosed in the mink (online Technical Appendix Table).

The 4 samples positive for the novel HEV variant were collected during 2008–2011 from herds across Jutland, Denmark, with a minimum distance of 80 km between the herds. The 4 PCR products obtained by the nested PCR, covering a region of 261 bp of the *RdRp* gene, were cloned and sequenced (GenBank accession nos. KC802090, KC802091, KC802092, and KC802093). The sequences were 98%–100% identical, with only 1 nonsynonymous mutation, resulting in a neutral amino acid change from isoleucine to valine (Technical Appendix Figure). The high homology in this region is not surprisingbecause the gene encodes the RNA polymerase. We initially tried to uncover a larger fragment by primer walking, but the limited amount of material prohibited this.

On the basis of the 261-bp fragment, we analyzed the phylogenetic relationship of this novel mink HEV variant to variants found in other animals (Figure). The mink HEV variant clustered with HEV variants found in ferrets and rats, which grouped in a separate branch that was clearly distinct from other previously described HEV variants. At nucleotide level, the mink HEV variant was ≈65% identical to the closest classical HEV genotype (G3 and G4) and 76% and 69% identical with ferret and rat HEVs, respectively. At the amino acid level, the homologies were more pronounced, showing ≈87% and ≈78% identity with ferret and rat HEV variants, respectively. The grouping of the HEV reference sequences in the analysis was identical with results of previously performed phylogenetic analysis on full-length sequences (*12*).

**Figure:**
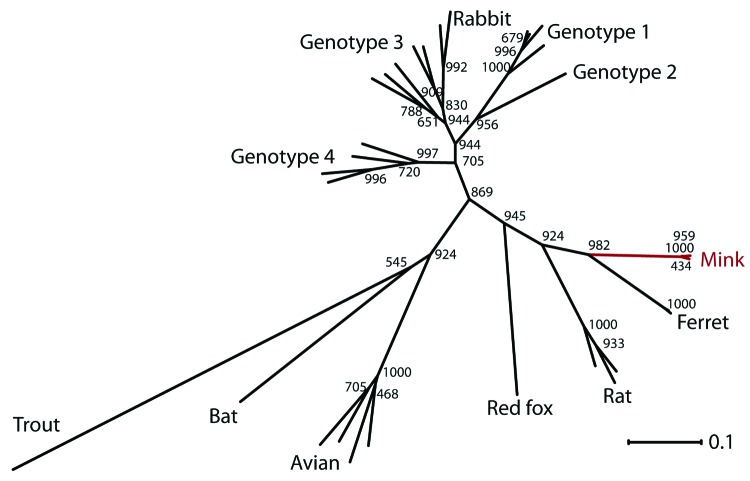
Phylogenetic tree showing the relationship between the novel mink hepatitis E virus (HEV), other HEV variants, and the 4 known HEV genotypes based on 261 bp of the *RdRp* gene. The CLC Main Workbench software (CLC bio, Aarhus, Denmark) was used for the phylogenetic analysis. Alignments were made by using MUSCLE algorithm (www.drive5.com/muscle/) and phylogenetic tree was made by using distance-based method with the neighbor-joining algorithm and bootstrap value of 1,000. Phylogenetic analysis with other methods showed similar results. [Q: Scale bar indicates what?]

## Conclusions

We detected a variant of HEV in 4 farmed mink from 4 geographically distinct locations in Denmark during a 3-year period, which indicates that the virus has been circulating among mink. Phylogenetic analysis showed that the virus was clearly distinct from, but closely related to, ferret and rat HEV variants recently reported from Germany and the United States (*6*,*7*,*13*).

It has not been possible to infect primates with rat or avian HEV variants (*13*,*14*). Thus, because of the phylogenetic resemblance of mink HEV with these nonzoonotic HEV variants, there are no indications that mink HEV can infect humans, although no human samples have been tested specifically for this virus. The zoonotic potential of HEV has been documented only in the case of G3 and G4, which were not found in mink. However, considering the relatively high HEV seroprevalence in humans, the possibility of other variants being zoonotic and cross-reacting with HEV G1–G4 in serologic assays cannot be ruled out.

The mink in this study were from herds that had mink enteritis virus, hepatic lipidosis, Aleutian mink disease virus, and catarrhal enteritis, all factors that could explain the conditions of the mink infected with HEV (*15*). However, it cannot be ruled out that the mink HEV variant contributed to the clinical signs of the mink HEV-positive animals. To determine whether the virus is indeed capable of inducing clinical signs in mink, the animals need to be experimentally infectedd. However, the rat and ferret HEV variants induced almost no histologic signs in rats after experimental infection, and the ferrets were described as not showing overt clinical signs (*7*,*13*). So far, only chickens infected with aHEV and humans infected with HEV G1–G4 have been described as being clinically affected by HEV infections. The possibility exists that the HEV variants recently reported in a variety of different species, including the 1 reported here, could evolve into disease-causing pathogens in animals and possibly also humans.

Technical Appendix Development of a specific real-time reverse transcription PCR to screen samples for the novel hepatitis E virus (HEV) variant; characterization of the 4 HEV-positive samples; and alignment of the 4 mink positive for HEV variant sequences obtained by the nested PCR.
