# Supplementary material for: Hepatitis E Virus Variant in Farmed Mink, Denmark
Source: Emerg Infect Dis. 2013 Dec;19(12):2028–30. doi: 10.3201/eid1912.130614 (PMC3840851; doi:10.3201/eid1912.130614)
Supplement: Technical Appendix — Development of a specific real-time reverse transcription PCR to screen samples for the novel hepatitis E virus (HEV) variant; characterization of the 4 HEV-positive samples; and alignment of the 4 mink positive for HEV variant sequences obtained by the nested PCR. [file 13-0614-Techapp-s1.pdf]

Article DOI: http://dx.doi.org/10.3201/eid1912.130614

Hepatitis E Virus Variant in Farmed Mink, Denmark

Technical Appendix

nPCR

The nested PCR (nPCR) was performed as described by John et al. (*1*), except that in this study the primer concentrations were 10 µM in the first round and 100 µM in the second round of the PCR.

Real-Time RT-PCR

A real-time reverse transcription PCR (RT-PCR) that specifically detected this novel mink HEV variant was developed on the basis of the sequence obtained from the first positive sample achieved by the nPCR. The nPCR product of this positive sample was cloned in pCR4 vector using the TOPO TA cloning kit (Invitrogen, Nærum, Denmark), hereafter referred to as pMINK. The real-time RT-PCR was designed based on the Primer Probe Energy Transfer (PriProET) chemistry, which allowed a few mismatches within the probe. The primers and probe were synthesized at Eurofins MWG Operon (Ebersberg, Germany). The unlabeled forward primer Mink-Fw 5′-CCAGAATGGTGCTTCTATGGTGAT-3′ had a calculated T_m of 63.6°C. The labeled reverse primer Mink-Rev 5′-FAM-AATTGTTCTGCGAGCTATCAAACTC-3′ had a calculated T_m of 62.5°C. The labeled probe Mink-Probe 5′-GCCCACACCTGCCGGGTCTTTGAAAACGATTT-ATTO633-3′ had a calculated T_m of 75.0°C. The location of primers and probe can be seen in Technical Appendix Figure. The real-time RT-PCR was performed by using QIAGEN OneStep RT-PCR kit (QIAGEN, Hilden, Germany) in a total volume of 25 µL, including 4 µL extracted RNA, 100 nM Mink-Fw, 500 nM Mink-Rev, and 500 nM Mink-Probe. The assay was run on a Rotor-Gene Q real-time PCR cycler (QIAGEN) with the 72-tube rotor and channel settings at 470 nm for excitation and 660 nm for acquisition. The temperature profile was divided into 3 segments with a reverse transcription

(RT) step, a PCR cycle, and a melt analysis (MA). RT: 48°C for 30 min, 95°C for 15 min. PCR: 40 cycles of 94°C for 15 s, 55°C for 1 sec with signal acquisition, and then an additional 14 s at 55°C, 72°C for 20 s. MA: 95°C for 45 s followed by ramping the temperature from 45°C to 80°C in 1°C increments and a 7-sec halt and signal acquisition at each step. The gain-optimization was set to automatic before first acquisition on tube 2. Data analysis was performed on the Rotor-Gene Q Series Software ver. 2.0.2 (QIAGEN), and the quantitation threshold was set at 0.01. The PCR performance was tested by a standard curve performed on a 10-fold serial dilution of the constructed pMINK plasmid showing a PCR efficiency of 76% ($R^2 = 0.999$), an 8 $\log_{10}$ detection span and an approximately detection limit at cycle threshold (Ct) 39. The melt analysis showed a T_m of 72 ± 2°C across the dynamic range. The sensitivity was tested on a 10-fold serial dilution of a positive mink fecal sample and compared with the nPCR. The real-time RT-PCR detected the 10^{-3} dilution, whereas the nPCR detected only the 10^{-1} dilution. All mink samples were run in duplicates with the duplicate being a 1:10 dilution of the original RNA extraction to exclude the influence of PCR inhibition.

Results

An alignment of the 4 sequenced mink HEV strains and the position of the developed mink HEV real-time PCR can be seen in the Technical Appendix Figure. A summary of the routine diagnostics analysis and the real-time RT-PCR results for the 4 positive samples are summarized in the Technical Appendix Table. One mutation was found in the probe region (Technical Appendix Figure) of the real-time RT-PCR in sequence 1119 and 574, which was consistent with the lower temperatures of the melting analysis (Technical Appendix Table).

Reference

1. Johne R, Plenge-Bönig A, Hess M, Ulrich RG, Reetz J, Schielke A. Detection of a novel hepatitis E-like virus in faeces of wild rats using a nested broad-spectrum RT-PCR. J Gen Virol. 2010;91:750–8. PubMed http://dx.doi.org/10.1099/vir.0.016584-0

Technical Appendix Table. Characterization of the 4 patients with hepatitis E virus–positive samples*

Sample no.	Year	Sex	Age	Pathology	Histopathology	Diagnosis	C_t	MA
345	2008	F	–	Liver: yellow, enlarged	Liver: massive diffuse lipid vacuolization<br>Small intestines: diffuse catarrhal enteritis	Hepatic lipidosis<br>Catarrhal enteritis<br>MeV not tested	25.1	73.2
1092-4†	2010	F	5 mo	Liver: yellow	Liver: lipid vacuolization, stasis<br>Small intestines: catarrhal enteritis	Hepatic lipidosis<br>Catarrhal enteritis<br>MeV diagnosed in herd	32.3	73.7
1092-5*	2010	M	5 mo	Liver: enlarged, hemorrhage, yellow<br>Spleen: enlarged, hemorrhage	Liver: massive lipid vacuolization, stasis, hemorrhage<br>Small intestine: catarrhal enteritis	Hepatic lipidosis<br>Catarrhal enteritis<br>MeV diagnosed in herd		
1119	2010	M	5 mo	Enteritis	Liver: moderate to severe lipid vacuolization, stasis<br>Small intestine: dilatation of intestinal crypt cells, microabscesses squamous cells, shortening and fusion of intestinal villi	Hepatic lipidosis<br>MeV diagnosed in subject and in herd	26.8	68.8
574	2011	M	3 mo	No macroscopic changes	Liver: no abnormalities<br>Small intestine: diffuse catarrhal enteritis	Catarrhal enteritis<br>MeV diagnosed in herd	31.5	69.0

*C_t, cycle threshold; MA, melt analysis; MeV, mink enteritis virus.
†The fecal sample from 1092-4 and 1092-5 were pooled; hence only 1 C_t and MA value was recorded.

```
                                                      Mink-Fw
                                              |-----------------------> 
                         10        20        30        40        50        60
                ....|....|....|....|....|....|....|....|....|....|....|....|
Mink 345        ................G..........A................................
Mink 1092       ............................................................
Mink 1119       .....................C........A.................C...........
Mink 574        ..................A.........................................
Consensus nt    AGCCATTGAGAAGCGCATTATTGACCAGCTCCCAGAATGGTGCTTCTATGGTGATGCCTA
Consensus aa      A  I  E  K  R  I  I  D  Q  L  P  E  W  C  F  Y  G  D  A  Y

                                                            Mink-Probe
                                                 |-------------------------
                         70        80        90       100       110       120
                ....|....|....|....|....|....|....|....|....|....|....|....|
Mink 345        .............................................C..............
Mink 1092       .............................................C..............
Mink 1119       .............................................T..............
Mink 574        .............................................T..............
Consensus nt    TGCCATGCCTAAATTTGAAGCAGCAGTTGCCGGCGCCCACACCTGYCGGGTCTTTGAAAA
Consensus aa      A  M  P  K  F  E  A  A  V  A  G  A  H  T  C  R  V  F  E  N

                                  Mink-Rev
                -----> <------------------------|
                        130       140       150       160       170       180
                ....|....|....|....|....|....|....|....|....|....|....|....|
Mink 345        ............................................................
Mink 1092       ............................................................
Mink 1119       ............................................................
Mink 574        ............................................................
Consensus nt    CGATTTTTCTGAGTTTGATAGCTCGCAGAACAATTTTTCTTTGGGACTTGAATGTTTGCT
Consensus aa      D  F  S  E  F  D  S  S  Q  N  N  F  S  L  G  L  E  C  L  L

                        190       200       210       220       230       240
                ....|....|....|....|....|....|....|....|....|....|....|....|
Mink 345        ............................................................
Mink 1092       ............................................................
Mink 1119       ............................................................
Mink 574        ....................................T.......................
Consensus nt    TATGGCTGAGGCAGGTGTACCAGAATGGTTGTGGAAGCTGTACCATCTCATTCGGAGTGC
Consensus aa      M  A  E  A  G  V  P  E  W  L  W  K  L  Y  H  L  I  R  S  A

                        250       260
                ....|....|....|....|.
Mink 345        .....................
Mink 1092       .....................
Mink 1119       .....................
Mink 574        .....................
Consensus nt    TTGGGTGTTGCAAGCCCCTCA
Consensus aa      W  V  L  Q  A  P
```

Technical Appendix Figure. Alignment of the 4 positive mink hepatitis E virus variant sequences obtained by the nested PCR. Arrows indicate the primers and probe of the real-time reverse transcription PCR. The nonsynonymous mutation is highlighted.
